# Evaluating the Influence of the Lens Autofluorescence on Adaptive Optics Fluorescence Lifetime Imaging Ophthalmoscopy

**DOI:** 10.64898/2025.12.03.25341556

**Published:** 2025-12-04

**Authors:** Ruixue Liu, Xiaolin Wang, Yuhua Zhang

**Affiliations:** 1 Doheny Eye Institute, Pasadena, California, United States; 2 The Department of Ophthalmology, University of California, Los Angeles, California, United States

**Keywords:** Adaptive optics, fluorescence lifetime, retinal pigment epithelium, autofluorescence, metabolic imaging, photon statistics, TCSPC, retinal imaging

## Abstract

**Purpose:**

To assess the impact of lens autofluorescence on adaptive optics fluorescence lifetime imaging ophthalmoscopy (AOFLIO).

**Methods:**

Eighteen subjects (n = 18) aged 23 to 68 years with normal chorioretinal health and phakic lenses were imaged using a research-grade AOFLIO instrument. Retinal autofluorescence was excited with a pulsed diode laser (λ=473nm) and detected in two spectral channels (500–560 nm and 560–720 nm). AOFLIO images were acquired at locations from the foveal center to 10° nasal retina. Autofluorescence decay was modeled using bi-exponential and tri-exponential functions, with and without accounting for the early arriving of lens signal. The contribution of lens autofluorescence was evaluated in relation to age an

**Results:**

The tri-exponential model that accounted for the early arriving lens signal demonstrated superior conformity to the measured autofluorescence decay. The amplitude coefficient of lens contribution was 3 – 4%. However, this component accounted for 20 – 30% of photon-weighted signals and resulted in a 30 – 40% overestimation of the mean retinal fluorescence lifetime when uncorrected. The effect was more pronounced in the short spectral channel (p < 0.001) and negatively correlated with axial length (p = 0.049).

**Conclusions:**

AOFLIO is inherently affected by lens autofluorescence. Accurate correction of the lens component is essential to obtain reliable retinal autofluorescence lifetime measurements.

## Translational Relevance

Minimizing the impact of lens autofluorescence in AOFLIO ensures accurate assessments of metabolic status in the retina and retinal pigment epithelium at the cellular level.

## 1. Introduction

Fluorescence lifetime imaging ophthalmoscopy (FLIO) is an emerging imaging modality for assessing the metabolic function of the retina and the retinal pigment epithelium (RPE) by measuring the autofluorescence lifetime of endogenous fluorophores. Under one-photon excitation, these fluorophores include lipofuscin, melanolipofuscin, and melanosome within the RPE, enzymes containing flavin adenine dinucleotide (FAD) or flavin mononucleotide (FMN) located in mitochondria, advanced glycation end products associated with conditions such as diabetic retinopathy, as well as collagen, and elastin in the Bruch’s membrane^1–4^. These fluorescent molecules arise as byproducts of cellular metabolism and oxidative stress, or function as coenzymes in energy production, carrying out critical metabolic functions vital to retinal health. Alterations in their fluorescence lifetimes have been found to be associated with aging and the development of retinal diseases^5–10^. Thus, accurate measurement of autofluorescence lifetimes can potentially detect the functional changes in early stages of retinal pathology. FLIO has been demonstrated for studying retinal and neurodegenerative diseases, including age-related macular degeneration, diabetic retinopathy, retinitis pigmentosa, Stargardt disease, and Alzheimer’s diseases^11–19^.

Albeit its success in clinical applications, the accuracy of FLIO has been found to be substantially impacted by autofluorescence from the human lens^20,21^, which has a much longer lifetimes (on the nanosecond scale) compared to retinal fluorophores (on the order of several hundred picoseconds). The lens’ autofluorescence resulted in an artificial prolongation of retinal autofluorescence lifetimes in FLIO, even with a confocal imaging mechanism^20,22–24^. To mitigate this artifact, imaging strategies with dual-point confocal scanning and aperture stop separation have been proposed to suppress lens fluorescence^23,25^, and mathematical models incorporating the contribution of the lens’ autofluorescence were developed to improve FLIO’s fitting accuracy^26,27^.

Adaptive optics fluorescence lifetime imaging ophthalmoscopy (AOFLIO) represents a recent advancement that integrates adaptive optics (AO) to enhance confocal FLIO imaging, improving both lateral and axial resolutions^28–31^. We have developed an AOFLIO system and demonstrated in vivo assessment of RPE metabolic function at the cellular and subcellular levels.^32–35^ In this study, we evaluated the impact of lens autofluorescence on AOFLIO in human subjects of varying ages with normal chorioretinal health.

## 2. Methods

The study followed the tenets of the Declaration of Helsinki, complied with the Health Insurance Portability and Accountability Act of 1996, and was approved by the Institutional Review Boards at the University of California at Los Angeles. Written informed consent was obtained from all participants.

### 2.1 Subjects

The participants were recruited from the clinical registry of the Department of Ophthalmology of the University of California, Los Angeles. The inclusion criteria for a normal subject were age greater than 18, best-corrected visual acuity (BCVA) of 20/25 or better, refractive error within ±8 D spherical and ±3 D cylindrical, no history of ocular and systemic disease, no significant cataract. A self-report questionnaire was used to ensure participants were in chorioretinal health.

The enrolled subjects underwent multimodal ophthalmic imaging, including color fundus photography, fundus autofluorescence imaging (excitation, 488 nm; emission > 600 nm) using an ultra-widefield (UWF^™^) retinal imager (California, Optos, Inc., Marlborough, MA), and spectral-domain optical coherence tomography (SD-OCT, Spectralis, Heidelberg Engineering Inc., Franklin, MA). Prior to imaging, subjects’ pupils were dilated, and accommodation was paralyzed by instillation of topical tropicamide (1%) and phenylephrine hydrochloride (2.5%) to ensure optimal imaging conditions. A retinal specialist reviewed the images to ascertain retina health status. Axial length measurements were obtained using an ocular biometer (IOL Master; Carl Zeiss Meditec, Germany).

### 2.2 AOFLIO instrument

The AOFLIO instrument used in this study was reported elsewhere.^32–35^ For the readers’ convenience, we summarize the technical details here. The AOFLIO was developed based on an adaptive optics scanning laser ophthalmoscope (AOSLO)^36–39^, which operates with a confocal imaging mode using an infrared low coherence light source (Superluminescent diode HP 795, Superlum Ltd, Ireland, λ=795nm, Δλ=15nm). The AO system consists of a custom designed Shack-Hartmann wavefront sensor and a deformable mirror with 97 actuators.

The AOFLIO employs a pulsed diode laser with wavelength at 473 nm (BDS-SM-473, Becker & Hickl GmbH, Berlin, Germany) as the excitation source. The emitted autofluorescence was detected simultaneously in two spectral channels: a short spectral channel (SSC, 500–560 nm) and a long spectral channel (LSC, 560–720 nm). The arriving times of the autofluorescent photons to their excitations were recorded by a time-correlated single photon counting (TCSPC) module (SPC-180nx, Becker & Hickl GmbH, Berlin, Germany) at a frequency of 40 MHz. AOFLIO and AOSLO were acquired through a pupil diameter of 6 mm. The confocal pinholes have a diameter of 150 μm, which corresponds to 5.8 Airy disk diameters (ADD) for the LSC and 7.0 ADD for the SSC, resulting in a lateral resolution of approximately 2 μm and an axial resolution of approximately 70 μm. Retinal images were acquired over a field of view of 1.2° × 1.2° and digitized by 512 × 512 pixels at a frame rate of 15 frames per second. For each subject, AOFLIO images were taken at 4 – 5 retinal locations from the foveal center extending to 10 degrees nasally in the horizontal retinal median.

The TCSPC module measured the time interval between the arriving autofluorescence photon and the closest excitation laser pulse and also marked the elapsed time since the recording began. Autofluorescence photons were allocated to a two-dimensional image matrix based on the line- and frame-synchronizations signals, and the AOSLO pixel clock. To accurately compensate for ocular movements, non-interpolative desinusoidal and substrip-based non-interpolative image registration algorithms^40^ were applied to precisely assign the autofluorescence photons to the corresponding spatial pixel location, ensuring accurate construction of the fluorescence lifetime histogram on each pixel.

### 2.3 Data Analysis

Retinal autofluorescence lifetime data were analyzed using SPCImage software (Version 8.8, Becker & Hickl GmbH, Berlin, Germany). Statistical analyses were carried out using MATLAB (MATLAB R2024a, The MathWorks, Inc., Natick, MA) statistical toolbox.

We compared three fluorescence decay models^41^, including

1. A tri-exponential model accounting for the early arriving lens signal with a time shift (S3-exp):
$$f(t)=a_{1}e^{-t/\tau 1}+a_{2}e^{-t/\tau 2}+a_{3}e^{-\left(t-\tau_{\text{shift}}\right)/\tau 3}$$
2. A tri-exponential model without accounting for the early arriving lens signal (3-exp):
$$f(t)=a_{1}e^{-t/\tau 1}+a_{2}e^{-t/\tau 2}+a_{3}e^{-t/\tau 3}$$
3. A conventional bi-exponential model (2-exp): $f(t)=a_{1}e^{-t/\tau 1}+a_{2}e^{-t/\tau 2}$

where f(t) represents the fluorescence intensity at time t, $a_{i}$ denotes the amplitude of the fluorescent component, $\tau_{i}$ the lifetime of the fluorescent components, and $\tau_{\text{shift}}$ the time shift parameter for the lens signal.

The mean autofluorescence lifetime was calculated as follows:

$$\tau_{m}=\frac{\sum a_{i}\tau_{i}}{\sum a_{i}}$$

For the S3-exp model, the mean retinal autofluorescence lifetime was estimated using the first two components ($\tau_{1},\;\tau_{2}$) and their amplitudes ($a_{1},\;a_{2}$):

$$\tau_{m12}=\frac{a_{1}\tau_{1}+a_{2}\tau_{2}}{a_{1}+a_{2}}$$

The contribution of each component was assessed using:

$$q_{i}=\frac{a_{i}\tau_{i}}{\sum_{j=1}^{n}a_{j}\tau_{j}}$$

Before model fitting, each model independently optimized the instrumental response function (IRF) to ensure an optimal fitting. The autofluorescence decay was fitted over a binning of 13 × 13 pixels across the AOFLIO image area using a moving pixel binning scheme. To ensure reliable curve fitting, only regions with photon counts greater than 2,000 were included in the analysis. In this study, the photon counts per histogram in the selected regions ranged from 2,000 to 20,616 in the LSC and from 2,000 to 9,363 in SSC.

The optimal temporal shift parameter in the S3-exp model was determined by the chi-square (X^2^) value. The one corresponding to the minimum X^2^ was considered as the optimal shift.

Statistical analysis was performed to evaluate differences among the three models using one-way analysis of variance (ANOVA), followed by Tukey’s post-hoc tests for pairwise comparison. Linear mixed regression modeling and two-way ANOVA were performed to evaluate the effects of age and imaging location (retinal eccentricity, expressed in degrees) on fluorescence lifetime parameters ($\tau_{3}$ and $a_{3}$) derived from the S3-exp model. Participants were divided into two age groups. Specifically, regression analyses were executed individually within each age group to examine trends with imaging location. ANOVA was used to test the significance of factors (degree and age group) in each model, and statistical significance was determined by the p-values from these tests. Pearson correlation analysis was performed to evaluate the relationship between axial length and temporal shift parameter. The strength of correlation was interpreted as follows: weak correlation (|r|<0.3), moderate correlation (0.3≤|r|<0.7), and strong correlation (|r|≥0.7). A p value < 0.05 was considered statistically significant.

## 3. Results

### Subject characteristics:

A total of 18 subjects were enrolled, with an age range from 23 to 68 years, including 7 males and 11 females. Refraction error: 0.03 ± 1.38 D (ranging from −7.0 D to 1.75 D). Axial length: 24.45 ± 1.26 mm. Participants were studied in two age groups: the younger group (n = 9), aged 32.4 ± 4.9 years ranging from 23 to 39 years; the older group (n = 9), age of 59.6 ± 5.1 years ranging from 53 to 68 years.

### 3.1 The S3-exp model demonstrated superior conformity to the autofluorescence decay

**Fig. 1:**
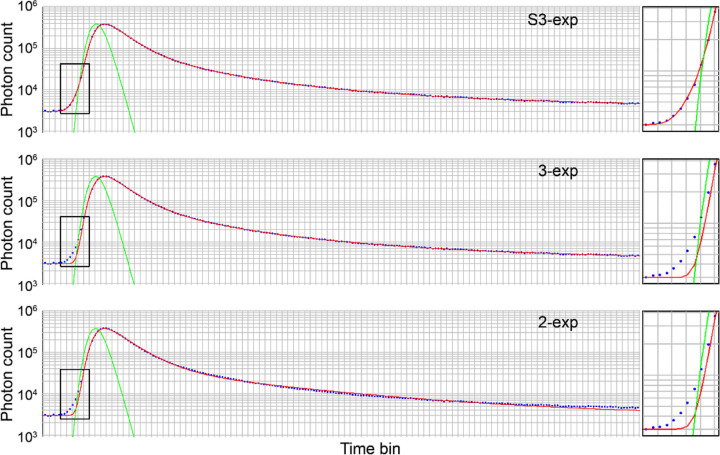
Modeling the fluorescence decay measured by the AOFLIO using bi-exponential (2-exp), tri-exponential with (S3-exp) and without (3-exp) accounting for lens signal. The boxes show magnified conformity of the AOFLIO measurements with the fitting curve in the rising edge zone. Blue dots are AOFLIO measurements of the photon numbers in the subsequent time bins, representing the time difference between the autofluorescence photons and the excitations. Red lines represent the fitted curves. Green lines indicate the instrument response function.

The superior fitting accuracy of S3-exp model is demonstrated by the high conformity of the measurement data with the photon decay curve, especially in the rising edge zone. The S3-exp model presented the minimum discrepancy versus the 2-exp and standard 3-exp models (Fig. 1).

**Fig. 2:**
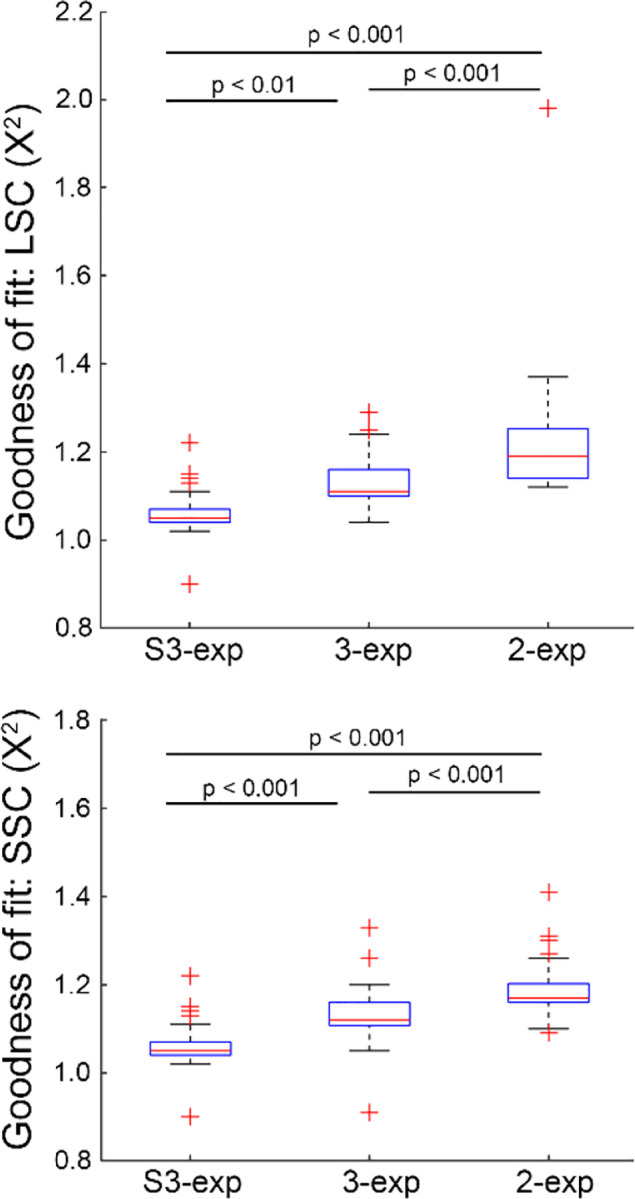
Comparison of the goodness-of-fit (X^2^) using bi-exponential (2-exp), tri-exponential with (S3-exp) and without (3-exp) accounting for early lens signal for estimating the autofluorescence lifetime in the long and short spectral channels.

As shown in Fig. 2, in the LSC, the mean X^2^ was 1.065 for the S3-exp model, compared to higher values of 1.131 and 1.230 for the standard 3-exp and 2-exp models, respectively. In the SSC, the S3-exp model achieved a mean X^2^ of 1.062, outperforming the standard 3-exp (X^2^ = 1.135) and 2-exp (X^2^ = 1.191) models. One-way ANOVA confirmed significant overall differences among these models (p < 0.0001 for both channels). Tukey’s post-hoc analysis of pairwise differences revealed the distinct superiority of the S3-exp model.

### 3.2 The time shifts in the S3-exp model is correlated with the eye’s axial length

**Fig. 3:**
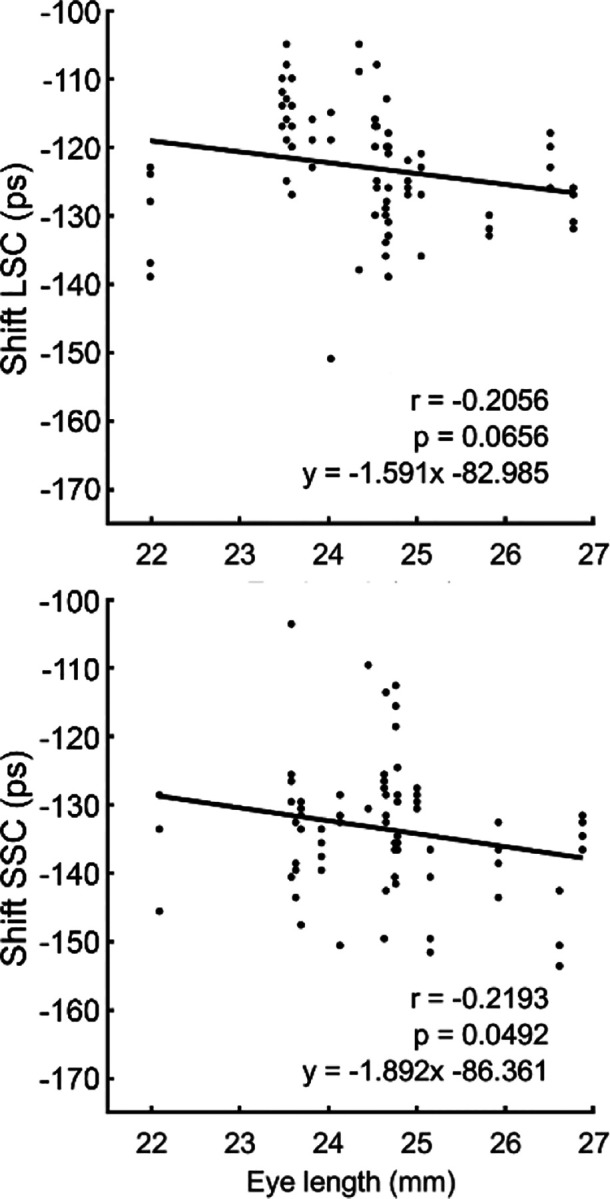
The time shifts in the tri-exponential model accounting for the early arriving of the lens signal versus the axial length of the eye in the long spectral channel (LSC) and short spectral channel (SSC).

In the LSC, the time shift was −122.0 ± 8.6 ps; in the SSC, it was −132.9 ± 9.8 ps. These values varied with the axial length. A negative correlation was observed in the LSC, but it did not reach statistical significance (r = −0.206, p = 0.066). In contrast, the negative correlation became significant in the SSC (r = −0.219, p = 0.049), Shown in Fig. 3. A negative shift value indicates that autofluorescence from the lens arrives earlier than that from the retina. A smaller negative value corresponds to a greater time difference between lens and fundus autofluorescence signals, particularly significant in the SSC.

### 3.3 The Lens Autofluorescence versus Age and Retinal Eccentricity

**Fig. 4:**
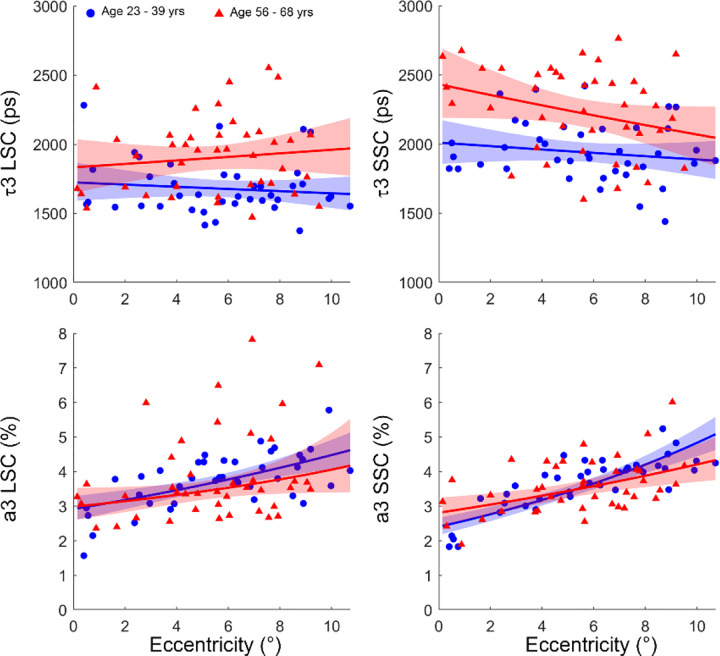
Lens autofluorescence estimated in the long (LSC) and short spectral channel (SSC) across different ages and retinal eccentricities by S3-exp model.

ANOVA analysis revealed a significant age dependency of the fluorescence lifetime of the third component ($\tau_{3}$) of the S3-exp model in both channels (all p ≤ 0.001, Fig. 4 top row); but the weight of this component was not influenced by age in either channel (LSC: p=0.449; SSC: p=0.934; Fig. 4 bottom row). Meanwhile, $\tau_{3}$ was found independent of retinal eccentricity (LSC: p = 0.930; SSC: p = 0.727). However, $a_{3}$ exhibited a significant dependence on the retinal eccentricity in both channels (LSC: p=0.015; SSC: p<0.001).

### 3.4 The weight of the Lens Autofluorescence in AOFLIO

When estimated using the S3-exp model, the amplitudes of the lens autofluorescence ($a_{3}$) were 3.8 ± 1.1% in the LSC and 3.6 ± 0.8% in the SSC. In contrast, evaluation with the standard 3-exp model yielded $a_{3}$ values of 3.1 ± 1.1% in the LSC and 2.7 ± 0.8% in the SSC. Despite the small weight of the third component to the mean fluorescence lifetime, it accounts for 19.4% to 30.0% of the total photons (Table 1), comparable to the values measured through pseudophakic eyes without autofluorescent lenses using the clinical FLIO instrument.

**Fig. 5:**
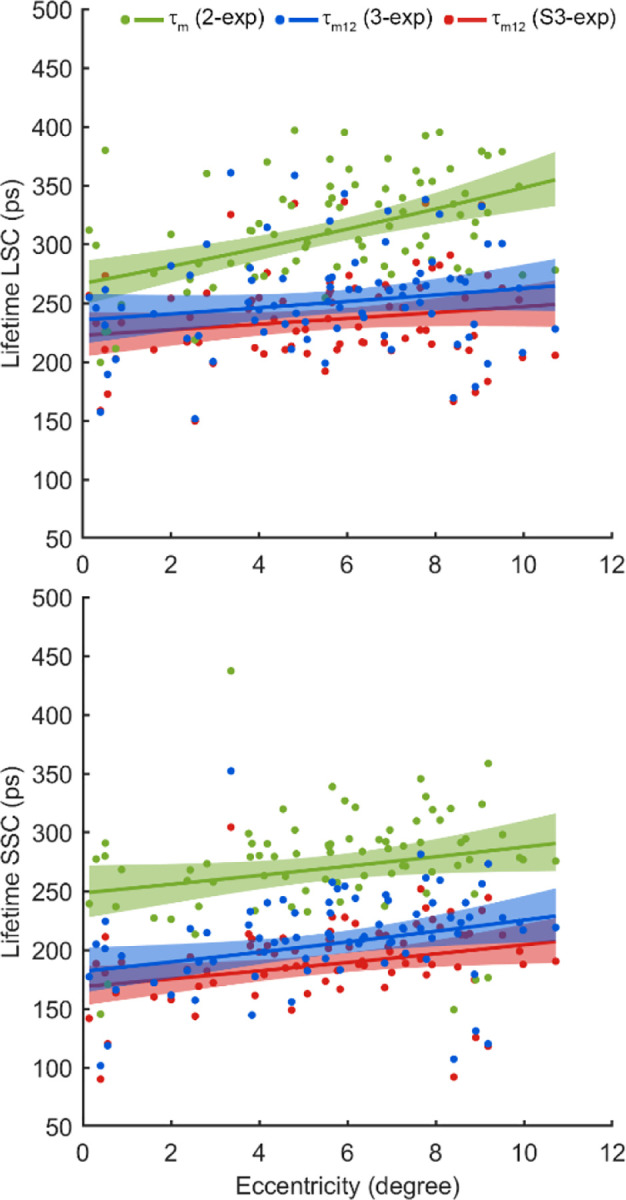
Mean retinal autofluorescence lifetimes estimated using the bi-exponential (2-exp), tri-exponential models with (S3-exp) and without (3-exp) $\tau_{\text{shift}}$ accounting for the early arriving of the lens signal in both the long spectral channel (LSC) and short spectral channel (SSC) across the retinal eccentricities.

The third component imposed a significant impact on the estimation of the mean retinal autofluorescence lifetime (Fig. 5). Specifically, the mean retinal lifetime ($\tau_{m}$) derived from the 2-exp model was substantially prolonged relative to the $\tau_{m12}$values obtained by the standard 3-exp and S3-exp models, due to the lens fluorescence influence. In the LSC, $\tau_{m}$ from the 2-exp model (312 ps) was prolonged by 22.8% compared to 3-exp $\tau_{m12}$ (254.0 ps, p < 0.0001) and by 30.2% compared to S3-exp $\tau_{m12}$ (239.6 ps, p < 0.0001). Similarly, in the SSC, the $\tau_{m}$ of the 2-exp model (273.5 ps) was prolonged by 30.7% compared to 3-exp $\tau_{m12}$ (209.2 ps, p < 0.0001) and by 42.9% compared to S3-exp $\tau_{m12}$ (191.4 ps, p < 0.0001). Moreover, when comparing the standard 3-exp and S3-exp models, the 3-exp model exhibited significantly prolonged lifetimes, as it did not account for the early arrival of lens fluorescence. The $\tau_{m12}$of the 3-exp model was prolonged by 5.7% in the LSC (254.0 ps vs. 239.6 ps, p < 0.001), and by 8.5% in the SSC (209.2 ps vs. 191.4 ps, p < 0.001).

## 4. Discussion

In this study, we evaluated the lens’ autofluorescence impact on AOFLIO measurement using 3 mathematical functions that model the fluorescence decay with 2 and 3 exponential components. We have demonstrated that AOFLIO effectively reduces the influence of crystalline lens autofluorescence. However, the impact of residual lens fluorescence signal on the measured retinal fluorescence lifetimes remains non-negligible. To obtain reliable and accurate retinal fluorescence lifetime, it is critical to remove lens contributions.

AOFLIO represents a significant advancement promising to assess the retinal and RPE metabolic function at the cellular level by compensating for the imaging light wave aberration caused by the optical defects of the human eye. It directly revealed individual RPE cells with the autofluorescence photons captured for measuring the fluorescence lifetime of the intrinsic fluorophores, demonstrating enhanced confocal imaging ability to reject out-of-focus signals. However, even with confocal imaging, clinical application and theoretical analysis have indicated that FLIO still collects autofluorescence emitted by the crystalline lens^23^. Thus, to measure the fluorescence lifetime accurately, the fluorescence decay must be appropriately modeled to minimize the influence of the lens autofluorescence. Our study showed that the conventional 2-exp and 3-exp models produced poor fits at the rising edge of the autofluorescence decay curve (Fig. 1), unable to reflect the photons that arrive earlier than expected from the retina. This finding agrees with previous FLIO studies^2,23,25^, which have shown that the lens fluorophores have long fluorescence lifetimes in the nanosecond range. Compared to autofluorescence emitted from the retina-RPE complex, autofluorescence from the lens arrives earlier due to the reduced light path. The S3-exp model, in which the third exponential component is temporally advanced by a time shift, provided optimal and biophysically interpretable modeling of this early component and the overall autofluorescence decay (Fig. 2). This model accurately reflected the early arrived lens signal by incorporating a temporal shift parameter relating to the spatial separation between the lens and the retina, which was found to be negatively correlated with the axial length of the eye (Fig. 3).

With the S3-exp model, we found that the fluorescence lifetime of the third component ($\tau_{3}$) measured by AOFLIO increased significantly with age in both SSC and LSC (all p < 0.001) (Fig. 4, top panels). However, the amplitude $\left(a_{3}\right)$, although varied with retinal eccentricity, did not exhibit significant change with age (Fig. 4, bottom panels). These results mirror previous FLIO measurements in normal aging eyes, where lens-related components increase with age^20,23,43^. The significant increase in the amplitude of the third component may also be related to differences in the angular collection efficiency or localized changes in lens transmission or backscattered light from different fundus locations. Specifically, when AOFLIO acquired images at increased retinal eccentricity, the lens became tilted, resulting in a longer optical path length and consequently an increased number of autofluorescence photons.

Our results showed that AOFLIO effectively suppressed the weight of the lens signal compared to conventional FLIO. Adaptive optics increased the number of autofluorescence photons detected from the retina-RPE complex through the confocal pinhole and enhanced spatial rejection of out-of-focus signals. This effect is reflected in the measured $q_{3}$ values of the third exponential component. In our dataset, the $q_{3}$ contribution in AOFLIO was 22.7% (LSC) and 29.0% (SSC), respectively. These values closely aligned with those reported for pseudophakic eyes in FLIO studies^23,42^, where the natural lenses were surgically removed. In contrast, $q_{3}$ values in FLIO phakic^42^ can be in a range from 20% to 50% in LSC, and 26–74% in SSC (Table 1). This comparison indicates that AOFLIO measurement matches the suppression level achieved through pseudophakic lenses, which are not fluorescent.

A small amplitude of the third component can lead to large errors in fluorescence lifetime estimation. The amplitude $a_{3}$ of the lens-related component in our dataset was within a range of 3% – 4% in both SSC and LSC, which agrees with previous AOFLIO studies that reported $a_{3}<5\%$ in healthy phakic eyes^31^. However, its contribution to total photon-weighted lifetime is disproportionately large, ranging from 20% to 30% in our data and causing overestimation of the fluorescence lifetime by 30–40% if uncorrected (Fig. 5). These results emphasize that even a small intrusion of the lens signal with long fluorescence lifetime can substantially bias retinal fluorescence lifetime estimation.

Across multiple metrics, the lens effects were greater in the SSC than in the LSC. First, the $q_{3}$ values were consistently higher in SSC (Table 1). Second, fluorescence lifetime differences between 2-exp and S3-exp models were more pronounced in SSC. Third, the correlation between axial length and shift value, a proxy for temporal separation between lens and fundus, was statistically significant in SSC (r = −0.219, p = 0.049) but not in LSC (r = −0.206, p = 0.066) (Fig. 3). These results align with prior FLIO studies reporting that SSC is more sensitive to lens fluorescence due to the emission spectra of major lens fluorophores such as AGEs and 3-hydroxykynurenine glucoside (3-OHKG), which fluoresce predominantly between 500–600 nm^20,22,43^.

Strengths of our study are AOFLIO acquired from a group of well characterized normal phakic eyes, a novel AOFLIO instrument with robust adaptive optics compensation for human ocular aberrations, a systematic evaluation of the autofluorescence lifetime estimated using multiple mathematic models, and comparative analyses across different age groups and retinal eccentricities. Limitations of this study include a relatively small sample size and the absence of subjects with pseudophakic lenses. Additionally, all measurements were conducted using a fixed confocal pinhole size.

Despite these limitations, our analysis suggests that AOFLIO has achieved near-pseudophakic suppression of lens signal. Future improvements are possible. Smaller confocal pinholes may further enhance axial sectioning and reject out-of-focus signals. Moreover, advanced imaging system design with ring-shaped aperture or dual-point scanning may be incorporate to minimize lens fluorescence^25^. Additionally, subject-specific shift calibration based on ocular biometry (e.g., axial length, lens thickness) could improve model accuracy.

In conclusion, AOFLIO is affected by the inherent autofluorescence emitted by the natural lens. A computational model with 3 exponential functions and compensating for the time difference of the lens signal is necessary for accurate measurement of the retinal autofluorescence lifetime. Minimizing the influence of lens autofluorescence is crucial in studies aiming to accurately measure the metabolic status of the retina, especially in aging populations and disease conditions where subtle changes in autofluorescence lifetime may serve as early indicators of pathology.

## Figures and Tables

**Table 1:** Photon ratio of the third exponential component in phakic and pseudophakic eyes

	3-exp		S3-exp	
Studies	LSC $q_{3}$ (%)	SSC $q_{3}$ (%)	LSC $q_{3}$ (%)	SSC $q_{3}$ (%)
**Phakic eyes (this study)**	**19.4 ± 5.0**	**24.1 ± 4.3**	**22.7 ± 3.6**	**29.0 ± 2.7**
Phakic eyes^42^	20 ~ 50	26 ~ 74		
Pseudophakic eyes^42^	22 ~ 46	22 ~ 46		
Pseudophakic eyes^23^	21.6 ± 2.9	24.7 ± 3.2		
Cataractous eyes 1^23^	28.4 ± 6.0	51.3 ± 10.3		
Cataractous eyes 2^27^	25.4 – 37.3	44.9 – 56.5	25.6 – 38.8	48.7 – 59.9
Cataractous eyes 3^20^			29.5 ± 9.9	54.2 ± 10.6

## Data Availability

All data produced in the present work are contained in the manuscript.
